# A Treatise on Diseases of the Air-Passages: Comprising an Inquiry and Treatment of Bronchitis, Chronic Laryngitis, Clergyman’s Sore-Throat, Etc.

**Published:** 1859-07

**Authors:** 


ART. IV.—*A Treatise on Diseases of the Air-Passages: comprising an Inquiry into the History, Pathology, Causes, and Treatment of those Affections of the Throat called Bronchitis, Chronic Laryngitis, Clergyman's Sore-Throat, etc. etc.* By HORACE GREEN, M.D., LL.D., President of the Faculty, and Emeritus Professor of the Theory and

Practice of Medicine in the New York Medical College, etc. Fourth Edition, revised and enlarged, with an Appendix. 8vo., pp. 348. New York: Wiley & Halsted. 1858.

IN 1816, in a work entitled *Surgical Observations; being a Quarterly Report of Cases in Surgery*, Sir Charles Bell reported several cases of disease of the respiratory passages, which were successfully treated by topical applications. One of these cases was that of a young woman whose glottis was extensively ulcerated. When Sir Charles first saw her, she was sitting up in bed, breathing with a "harsh, sawing sound;" her dyspnœa was so great as to threaten suffocation, and she spoke with difficulty and in whispers. "Having ascertained," writes Bell, "by putting my finger over the root of the tongue into the glottis, that it was rough and irregular with ulceration, I proposed to touch the surface with the *argentum nitratum*. It was considered hazardous, but something was necessary, and I was confident that the application would allay irritation. I made a small pad of lint, and attached it to the ring of a catheter-wire, and bent the wire so as to pass over the root of the tongue and epiglottis; I dipped the lint in a solution of twenty grains of the caustic to half an ounce of water, and touched the glottis with it in this manner. With the fingers of my left hand I pressed down the tongue, and stretched the forefinger over the epiglottis; then, directing the wire along my finger, I removed the point of the finger from the glottis, and introduced the pad of lint into the opening, and pressed it with my finger. On withdrawing the lint, instead of coughing, she began to speak more audibly than usual, and had neither cough nor spasm from this rough operation. I repeated the application four times, and her breathing was sensibly better when I left her." These applications were continued for many days, and the patient finally restored to health.

In 1837, Trousseau and Belloc prescribed and employed topical medications in chronic diseases of the larynx. Their work was translated into English by Dr. Warder, of Cincinnati, in 1839. They used solutions of nitrate of silver, corrosive sublimate, sulphate of copper, and nitrate of mercury. Of all these, however, they preferred the nitrate of silver, on account of the efficacy and rapidity of its action. The solution which they found most efficacious in the treatment of laryngeal disease, consisted of two drachms of the nitrate to an ounce, or, sometimes, to a half ounce of distilled water. This solution was applied to the epiglottis and upper part of the larynx, sometimes by means of a small, round piece of sponge attached to the bent end of a whalebone rod, and sometimes by employing a small silver syringe, having a long curved tube. This instrument being filled to one-fourth of its capacity, was carried beyond the epiglottis, and the solution forcibly discharged into the opening of the larynx.

In 1838, Dr. Johnson, the editor of the *Medico-Chirurgical Review*, suggested to Dr. Horace Green, who was at that time in London, the propriety and probable utility of making applications below the epiglottis in the treatment of chronic laryngeal disease. This suggestion was successfully put into execution by Dr. Green upon his return home to New York. In September, 1840, he brought this subject before the New York Medical and Surgical Society, and reported fifteen cases of laryngeal and bronchial disease successfully treated by the direct application of nitrate of silver solutions to the lining membrane of the larynx. Such a degree of skepticism on this subject was manifested at the time by a large proportion of the members, that, for many years, he refrained from bringing the matter again before the society. At length the subject was presented a second time to the society, by some of its members, who had themselves succeeded in applying remedial agents to the mucous membrane of the laryngeal cavity. But a majority of the members being still in doubt upon the subject, a committee was appointed to inquire more fully "into the practicability of making these topical applications to the surfaces of the larynx." The committee reported favorably; but the skeptics still asserted the impossibility of introducing a probang into the larynx. A fierce controversy ensued. Dr. Green attempted, in vain, to convince the doubters, by reminding them that coins and other foreign bodies are occasionally introduced into the larynx by accident; that Dessault long ago proposed the introduction of an elastic tube into the trachea, as a substitute for the operation of bronchotomy, in cases of suffocation arising from certain causes; that Ryland, in his work on *Diseases and Injuries of the Larynx and Trachea*, declares that a tube guided by a scientific hand could readily be made to pass into the trachea without irritation and without convulsive spasm; and, finally, that in several instances, when Baron Larrey passed a stomach-tube into the larynx instead of the œsophagus, the mistake was not discovered by any particular sensation about the glottis. Neither would they be convinced when our author demonstrated to them upon some of his patients the practicability of the operation; but, on the contrary, becoming more skeptical than ever, they made vigorous efforts to extinguish the obnoxious Dr. Green and his offensive probangs. Happily, persecution sooner or later arrests attention and raises up adherents for the persecuted. So it came to pass that the medical fraternity of New York split into two great factions, the *Probangers* and the *Anti-probangers.* Amid the incessant skirmishing of these Galenical Guelphs and Ghibellines, the undismayed and unextinguishable Dr. Green wielded his probangs more vigorously than ever.

Larynx after larynx was taken by surprise, trachea after trachea was invaded, and bronchial tubes shrank and grew white in the presence of the restless and resistless caustic sponge, until at last our author succeeded, in the teeth of all opposition, in establishing a bold and ingenious practice, "which, if judiciously employed," says Professor Bennett, of Edinburgh, "may form a new era in the treatment of pulmonary diseases."

From the *Appendix* to the present edition of the volume under consideration, we learn that since the publication of the first edition in 1846, many important treatises on the local treatment of laryngeal disease have been published in Europe. In 1848 appeared a work entitled *Dysphonia Clericorum, or Clergyman's Sore-Throat; its Pathology and Treatment.* By Dr. James Mackness, of London. Two years later, Dr. John Hastings, of the same city, published a *Treatise on Diseases of the Larynx and Trachea,* and their Treatment by the Local Application ot Caustics.

"Dr. Hastings expresses great confidence in the use of the nitrate of silver as a local remedy in the treatment of these affections; and he also details many cases of much interest, in which topical medication proved effectual in arresting the disease after other measures had failed. Under the head of 'Follicular Laryngitis,' Dr. Hastings alludes to a pathological condition of the larynx and trachea, which, as an independent affection, is very generally overlooked by the profession, or is considered the sequel—not, as it often is, the antecedent of tuberculosis. 'I am satisfied,' says Dr. Hastings, 'that cases presenting the morbid appearances in the pharynx and arch of the fauces just described, form but a small proportion of those denominated follicular laryngitis.' * * *

"'I have repeatedly met with cases in which the disease was confined to those parts and the back of the velum, where nothing more was required than to carry the solution of the nitrate of silver behind the uvula into the posterior nares, and over the pharynx and fauces, in order to remove a very troublesome cough; while in others, and by far the greater number, the disease exists in the larynx and trachea, the fauces and the pharynx at the same time presenting a healthy appearance.

"'Such cases are, generally, most puzzling to the practitioner. The patient is troubled with cough, the expectoration is muco-purulent, occasionally streaked with blood, to a considerable amount; pains are felt in the chest below the clavicles, he wastes a little, or he may not lose flesh. His chest is examined again and again, but no disease can be discovered; his mouth and throat are inspected without anything being found there to account for the symptoms; at length the disease is regarded as an obscure case of phthisis; he gets treated with sedatives, expectorants and cod-liver oil, until the ensuing winter, when all his former symptoms return in an aggravated degree, while as the warm season comes on they improve.'

"'Much pain and suffering might be spared in these cases were a stethoscopic examination of the windpipe resorted to, which in most cases would point out the nature, situation, and extent of the disease; and the practitioner would have that satisfaction in treating the case which an imperfect knowledge or an entire ignorance of it can never give.'

"Such cases are reported by Dr. Hastings as having been successfully treated by the repeated application of the sponge, saturated with a solution of the nitrate of silver, and which was 'carried down the windpipe,' he says, 'as low as the bifurcation of the bronchi.'"

In a paper read before the Edinburgh Medico-Chirurgical Society, by Dr. John Scott, one of the oldest and most distinguished physicians of Edinburgh, and afterwards published in the *Monthly Journal of Medical Science* for 1850, many interesting cases of laryngeal diseases, successfully treated by topical medication, are recorded. In 1851, M. Joubert, of France, published, in the *Recueil des Travaux de la Société Médicale de l'Indre et Loire*, a memoir on the local method of treating hooping-cough.

In 1852, Dr. Richard Payne Cotton, one of the physicians of the Brompton Hospital, published a work *On the Nature, Symptoms, and Treatment of Consumption.* After stating that he formerly disbelieved in the practicability and propriety of applying medicinal substances to the mucous membrane of the respiratory passages, Dr. Cotton says:—

"'I should here remark, that my own views upon this subject differ from those I formerly held and have even expressed; and that I owe this change to the kindness of Dr. Horace Green, of New York, the justly celebrated advocate of this treatment, who, during a recent visit to our metropolis, convinced myself and others not only of the possibility, but of the safety and usefulness of the practice.

"'I had long been in the habit of using a solution of nitrate of silver to the pharynx and upper surface of the epiglottis, by means of a soft brush, in all the early cases both of pharyngeal and laryngeal complication; and had frequently witnessed its good effects, not only upon the part to which it was immediately applied, but upon the laryngeal structures also, attributing it in the latter case to an action excited in the upper respiratory passages from continuity. But I had never ventured to apply anything directly to the larynx itself; not from misgiving as to its effects, but from apprehensions of its danger. For some months past, however, I have done so extensively in cases of chronic laryngitis, whether idiopathic or tubercular, and very frequently with marked success.

"'At the commencement of the laryngeal symptoms, a solution of the crystals of nitrate of silver, varying in strength from ten grains to half a drachm to the ounce of distilled water, passed, by means of the instrument recommended by Dr. Green, into the opening of the larynx, is often productive of great relief. I have known the voice regained, the irritable cough removed, and the tenderness and difficulty of swallowing dissipated entirely by it; indeed, I think we might almost speak of its *curative* effects, so far, at least, as the larynx is concerned, in some very early cases.'"

The next work in the chronological order, is that of Dr. S. Scott Alison, of Edinburgh, *On the Medication of the Larynx and Trachea*, published in 1853.

"'I had so frequently found,' says Dr. A., 'in the treatment of local disease and local complications, that many remedies were far more efficacious when applied immediately to the part affected or to its vicinity, than at a distance, that I was glad to learn that a sponge, loaded with the solution of the nitrate of silver, and affixed to a probang, could, not only without injury, but with manifest advantage, be passed through the glottis and the larynx down into the trachea.'

"In acute inflammation of the glottis, Dr. Alison has hesitated to apply the solution, lest 'the presence of the stimulant on the parts suffering from such attacks,' might aggravate the disease; but in chronic inflammations of the larynx, 'and of the upper portion of the trachea, the solution of the nitrate of silver, he observes, has in my hands, as in others, been very useful in bringing the disease to a conclusion; and where that has not been accomplished by reason of its dependence upon incurable disease of the lungs, it has almost invariably afforded very considerable relief, by rendering the cough less violent and frequent, and removing much of the tickling and uneasy sensations at the upper portion of the larynx. * * * 'In some cases of disease of the larynx and trachea,' he continues, 'in which the symptoms inclined to the suspicion that ulceration existed, the same local application of a solution of the nitrate of silver has been very useful.'"

Dr. Alison also recommends the employment of nitrate of silver in the treatment of the cough and irritation of the glottis, caused by the presence of tubercles in the lungs. He assures us that much comfort and benefit have been derived from its use, both when the tubercles have been crude and when they have become softened. The presence of undoubted cavities in the lungs, the breaking down of tubercles, and the expulsion of their débris, have not prevented this application from being decidedly useful.

In the same year, (1853,) Dr. J. Hughes Bennett, of Edinburgh, published his well-known *Treatise on the Pathology and Treatment of Pulmonary Tuberculosis and on the Local Medication of Pharyngeal and Laryngeal Diseases*. In this treatise, Professor Bennett declares that if the probang be properly prepared and the operation well performed, the sponge, saturated with the solution of nitrate of silver, may be rapidly thrust through the rima into the larynx, and frequently into the trachea. "I am persuaded," he says, "that on many occasions I have passed it pretty deep into the trachea, not only from the length of the probang, which has disappeared, but also from the sensations of the patient." In November, 1857, Dr. Bennett contributed to the *Edinburgh Medical Journal* a short article on *Injections of the Bronchi in Pulmonary Diseases*. In that article he says:—

"I have confirmed the statements made by Dr. Horace Green. I have introduced the catheter publicly in the clinical wards of the Royal Infirmary, in seven patients. Of these, five were affected with phthisis in various stages—one had chronic laryngitis, with bronchitis, and one chronic bronchitis, with severe paroxyms of asthma. In several other cases in which I attempted to pass the tube, it was found to be impossible—in some because the epiglottis could not be fairly exposed, and in others on account of the irritability of the fauces and too ready irritation of cough from pressure of the spatula.

"My experience of this treatment is as yet too limited to permit my saying anything of its permanent effects. In the case of bronchitis with asthma—a female, aged 24—I have now injected the lungs eleven times, at first throwing in ʒij of a solution of nitrate of silver, of the strength of ʒss of the crystallized salt to ℥j of distilled water, and latterly I have thrown in ℥ss of a solution of the strength of ℈ij to ℥j. She declares that no remedy has had such powerful effect in lessening the cough, diminishing the expectoration, or delaying the asthmatic paroxysms. She breathes and blows through the tube, when inserted four inches below the larynx, and I have been surprised at the circumstance of the injections not being followed by the slightest irritation whatever, but rather by a pleasant feeling of warmth in the chest, (some have experienced a sensation of coolness,) followed by ease to the cough, and a check for a time to all expectoration.

"I think it of importance that these facts should be known to the profession, as a homage justly due to the talents of a distinguished transatlantic physician, and with the view of recommending a practice which, if judiciously employed, may form a new era in the treatment of pulmonary diseases."

In the second edition of his *Clinical Lectures on the Principles and Practice of Medicine*, published in 1858, Dr. Bennett again takes occasion to speak of the great advantages to be derived from topical medication in the treatment of laryngeal diseases.

In 1854, Dr. Eben Watson put forth, at Glasgow, a work on the *Topical Medication of the Larynx in certain Diseases of the Respiratory and Vocal Organs.* To this gentleman belongs the honor of having been the first to employ local applications in the treatment of hooping-cough. His original paper on pertussis, in which he describes "a new method" of treating that disease, was read before the Medical Society of Glasgow, in 1849, and was first published in the *Edinburgh Monthly Journal* for December of that year. Five years later, after having treated many other cases by this method, Dr. Watson gives us the results of his experience in the work whose title we have just quoted. These results positively confirm the statements put forth by Dr. Green in 1846. Professor Robert B. Todd, Physician to King's College Hospital, London, has treated topically many cases of pharyngo-laryngeal and bronchial disease. In his "Clinical Lectures," recently published in the *London Medical Times and Gazette,* he recommends "the local application of a solution of nitrate of silver, (ʒss to the ℥j,) by means of a probang thrust behind the epiglottis down to the glottis," after the plan of Dr. Green. "The patient," he says, "can always tell whether the sponge enters the larynx or not, from the great irritation it excites when it passes into the glottis; and in the withdrawal of it, the operator feels a certain resistance, caused by the sponge being grasped by the muscles of the larynx, which resistance is not felt when it simply passes into the œsophagus."

In 1857, an interesting discussion, on the subject of the cauterization of the larynx and bronchi, took place in the Academy of Medicine of Paris.* A commission was appointed to investigate the subject, of which M. Trousseau was made chairman. A favorable report on the treatment of laryngeal affections by this method was made and unanimously adopted by the academy—the commission declaring that cauterization of the air-passages, in the treatment of disease, is not only practicable, but is of great utility. Finally, Professor Griesinger, of Germany, has declared himself an advocate of Dr. Green's method of treating certain diseases of the respiratory passages by topical medication.†

From this brief survey of the history and literature of this interesting subject, it will be seen that the practicability and utility of Dr. Green's method have, at length, after the lapse of nearly a quarter of a century, been sustained and verified by unquestionable medical authorities in Great Britain, France, and Germany. In our own country, on the contrary, the views of Dr. Green have gained but few adherents. In the city of his residence these views are even violently opposed by some of the oldest and most influential physicians, as the recent proceedings of the New York Academy of Medicine, relative to the death of Mr. Whitney, amply testify. We are neither acquainted with, nor have we ever seen any of the parties concerned in the disgraceful transaction to which we allude; we therefore speak disinterestedly when we say that, having read attentively the reports of these proceedings as published in the *American Medical Monthly* for February, 1859, and in other journals, we are constrained, from the evidence therein set forth, to regard the arraignment of Dr. Green before the Academy as an undignified, impolitic, and badly managed attempt to cast obloquy upon him, and to bring into disrepute his views concerning certain respiratory diseases, and the plan of treatment based upon these views.

---

* Gazette Médicale de Paris and L'Union Médicale de Paris, for August and September, 1857.

† Deutsche Klinik, quoted in the Gazette Hebdomadaire for May, 1858.

